# Biofunctional carboxymethyl chitosan hydrogels with nano-hydroxyapatite gradients accelerate bone defect healing

**DOI:** 10.1016/j.isci.2026.117200

**Published:** 2026-08-14

**Authors:** Xudong Li, Chengzhe Yang, Xin Li, Lanxin Li, Youbin Li, Tengyue Liu, Qiang Gao, Lanfeng Huang

**Affiliations:** 1 Department of Orthopedics, The Second Hospital of Jilin University, Changchun 130041, P.R. China

**Keywords:** bone regeneration, nano-hydroxyapatite, nHA, carboxymethyl chitosan, CMCS

## Abstract

Large bone defects remain a clinical challenge due to limited regenerative capacity. In this study, carboxymethyl chitosan (CMCS) hydrogels incorporating gradient nano-hydroxyapatite (nHA) were developed to enhance bone repair. The composite hydrogels exhibited improved mechanical properties, controlled degradation, and good biocompatibility. *In vitro*, nHA-containing hydrogels promoted bone marrow mesenchymal stem cell (BMSC) proliferation and osteogenic differentiation, as shown by increased alkaline phosphatase (ALP) activity, mineralization, and upregulation of runt-related transcription factor 2 (RUNX2), osteocalcin (OCN), and bone morphogenetic protein-2 (BMP-2). *In vivo*, implantation in a rat distal femoral condyle defect model showed enhanced new bone formation and improved trabecular structure, particularly in the 1% nHA group. These results suggest that gradient nHA-loaded CMCS hydrogels can effectively enhance osteogenesis and support bone regeneration.

## Highlights

• Graded nHA-loaded CMCS hydrogels promote BMSC osteogenic differentiation
• nHA enhances mineral ion release, viscoelasticity, and compressive stiffness
• 1% nHA balances scaffold processability, stability, and osteogenic bioactivity
• CMCS/GP/nHA hydrogels accelerate repair in a rat femoral condyle defect model

## Graphical abstract

**Figure:**
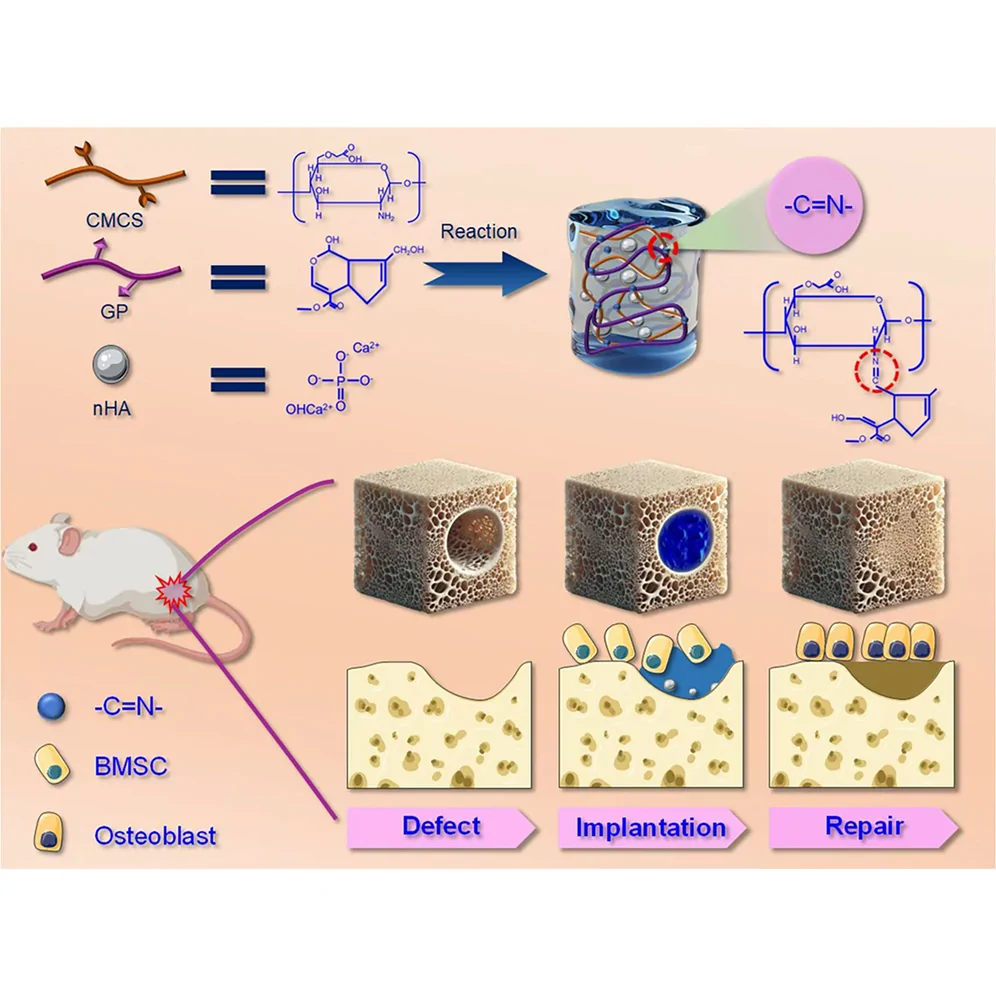

## Teaser

Materials science; Biomaterials

## Introduction

Bone tissue is an essential organ that provides mechanical support and enables body movement, and its homeostasis is maintained through a dynamic balance between bone formation and resorption.^1^^,^^2^^,^^3^^,^^4^ Mild injuries can be repaired naturally through a series of biological processes, including vascular infiltration, osteoblast proliferation, and extracellular matrix mineralization.^5^^,^^6^ However, severe trauma, tumor resection, infectious destruction, prosthesis loosening, and congenital disorders often result in bone defects that exceed the intrinsic regenerative capacity of bone.^7^^,^^8^^,^^9^ Such defects hinder biomechanical restoration and structural healing, leading to impaired limb function and compromised quality of life.^10^^,^^11^^,^^12^ Therefore, promoting efficient repair of large bone defects remains a major clinical challenge in orthopedics and regenerative medicine.^13^

Currently, bone grafting is the primary clinical intervention to address bone defects, including autografts, allografts, xenografts, and synthetic substitutes.^14^^,^^15^^,^^16^ Autografts, regarded as the “gold standard,” provide excellent osteogenic, osteoinductive, and angiogenic properties but are limited by donor site morbidity and restricted tissue availability.^17^^,^^18^ Allografts and xenografts can partially alleviate tissue shortage yet suffer from risks of immune rejection, pathogen transmission, and insufficient osteoinductive capability.^19^^,^^20^^,^^21^ Meanwhile, synthetic grafts struggle to simultaneously meet biological compatibility and mechanical stability requirements.^22^^,^^23^ These limitations underscore the urgency of developing advanced strategies for effective bone regeneration.^24^

Bone tissue engineering (BTE), which integrates cells, biomaterial scaffolds, and bioactive cues, has emerged as a promising therapeutic approach.^25^^,^^26^^,^^27^ By constructing a biomimetic extracellular microenvironment, BTE aims to regulate cellular behavior, promote vascularization, and drive new tissue formation to restore both structure and function of the defect site.^28^^,^^29^ An ideal scaffold should exhibit excellent biocompatibility and biodegradability, interconnected porous architecture to support cell proliferation and nutrient exchange, appropriate mechanical integrity, and intrinsic osteoinductive or osteoconductive properties.^19^^,^^30^^,^^31^^,^^32^^,^^33^ Hydrogels, especially those derived from natural polymers, have attracted tremendous attention due to their high water content, tunable physicochemical properties, and extracellular matrix-like characteristics.^34^^,^^35^^,^^36^ Importantly, bone regeneration is not governed solely by osteogenic differentiation but requires a highly coordinated coupling between osteogenesis and angiogenesis.^37^ Newly formed blood vessels provide oxygen, nutrients, osteoprogenitor cells, and paracrine signals that are essential for extracellular matrix deposition and mineralized tissue maturation. Therefore, an ideal bone-regenerative scaffold should not only promote osteogenic lineage commitment but also provide a microenvironment permissive for vascular ingrowth and tissue remodeling. In this context, porous natural polymer hydrogels may facilitate nutrient diffusion and cellular infiltration, while bioactive inorganic components such as nHA may modulate the local ionic microenvironment and thereby contribute to osteogenesis-associated vascular remodeling.

Chitosan (CS), a natural polysaccharide extracted from crustacean shells, shows biodegradability, antibacterial activity, and intrinsic tissue adhesiveness.^31^^,^^38^ However, its poor water solubility and limited mechanical strength hinder its application in bone regeneration.^39^ Carboxymethyl chitosan (CMCS), a structurally modified derivative of CS, exhibits excellent solubility, enhanced antioxidant properties, and favorable osteogenic potential, making it a promising hydrogel matrix for bone repair.^39^^,^^40^^,^^41^ In parallel, nano-hydroxyapatite (nHA), the major inorganic component of native bone, provides superior osteoconductivity and biological affinity, facilitating cell mineralization and enhancing scaffold biomechanics.^42^^,^^43^ Combining nHA with natural polymer hydrogels is therefore a widely adopted strategy to improve regenerative performance.^44^ However, most previously reported nHA/polymer composite hydrogels have relied on a fixed or homogeneous nHA content, which may not fully reflect the heterogeneous mineral microenvironment of native bone.^45^ Native bone exhibits spatial and compositional variations in mineral density, stiffness, and biological cues, and these gradients are important for regulating cell adhesion, mechanotransduction, osteogenic differentiation, and tissue remodeling. In addition, a single homogeneous nHA concentration may either provide insufficient osteoconductive stimulation at low loading levels or induce particle aggregation and network instability at excessive loading levels. Therefore, a graded nHA-loading strategy was adopted in this study to establish a composition-structure-function relationship and to identify an optimized balance among hydrogel processability, structural stability, mechanical reinforcement, and osteogenic bioactivity.

Moreover, hydrogel stability is crucial for maintaining scaffold integrity following implantation.^46^^,^^47^^,^^48^ Genipin (GP), a natural low-toxicity crosslinker, reacts with amino groups to form stable three-dimensional networks and possesses anti-inflammatory and antioxidative activities.^49^^,^^50^^,^^51^ Compared with commonly used synthetic crosslinkers such as glutaraldehyde, GP is more suitable for *in vivo* biomaterial construction.^49^^,^^50^

In this study, we designed a biomimetic CMCS-based hydrogel scaffold crosslinked with GP and engineered with graded nHA loading to enhance osteogenic bioactivity. We systematically evaluated its cytocompatibility and osteoinductive potential using bone marrow mesenchymal stem cells (BMSCs), and assessed its ability to facilitate bone repair in a standardized rat distal femoral condyle defect model. In addition, the potential contribution of CMCS/GP/nHA hydrogels to the osteogenesis-angiogenesis coupling microenvironment was discussed to provide a more comprehensive interpretation of their biological activity.

## Results

### Fabrication and physicochemical characterization of composite hydrogels

Composite hydrogels were successfully fabricated by GP crosslinking of CMCS followed by incorporation of nHA at different mass fractions (Figure S1A). Based on preliminary optimization, the maximum nHA concentration was set at 1%, because higher nHA contents led to reduced dispersion stability, visible precipitation, and difficulty in maintaining a homogeneous CMCS/GP precursor solution. These observations suggested that excessive nHA loading may interfere with uniform hydrogel network formation. Therefore, 1% nHA was selected as the highest formulation in the present study to balance mineral bioactivity and hydrogel processability. All resulting hydrogels displayed a characteristic dark blue color, indicative of the stable chromophore formed through the reaction between GP and amino groups of CMCS, confirming efficient crosslinking and network formation.

**Figure 1:**
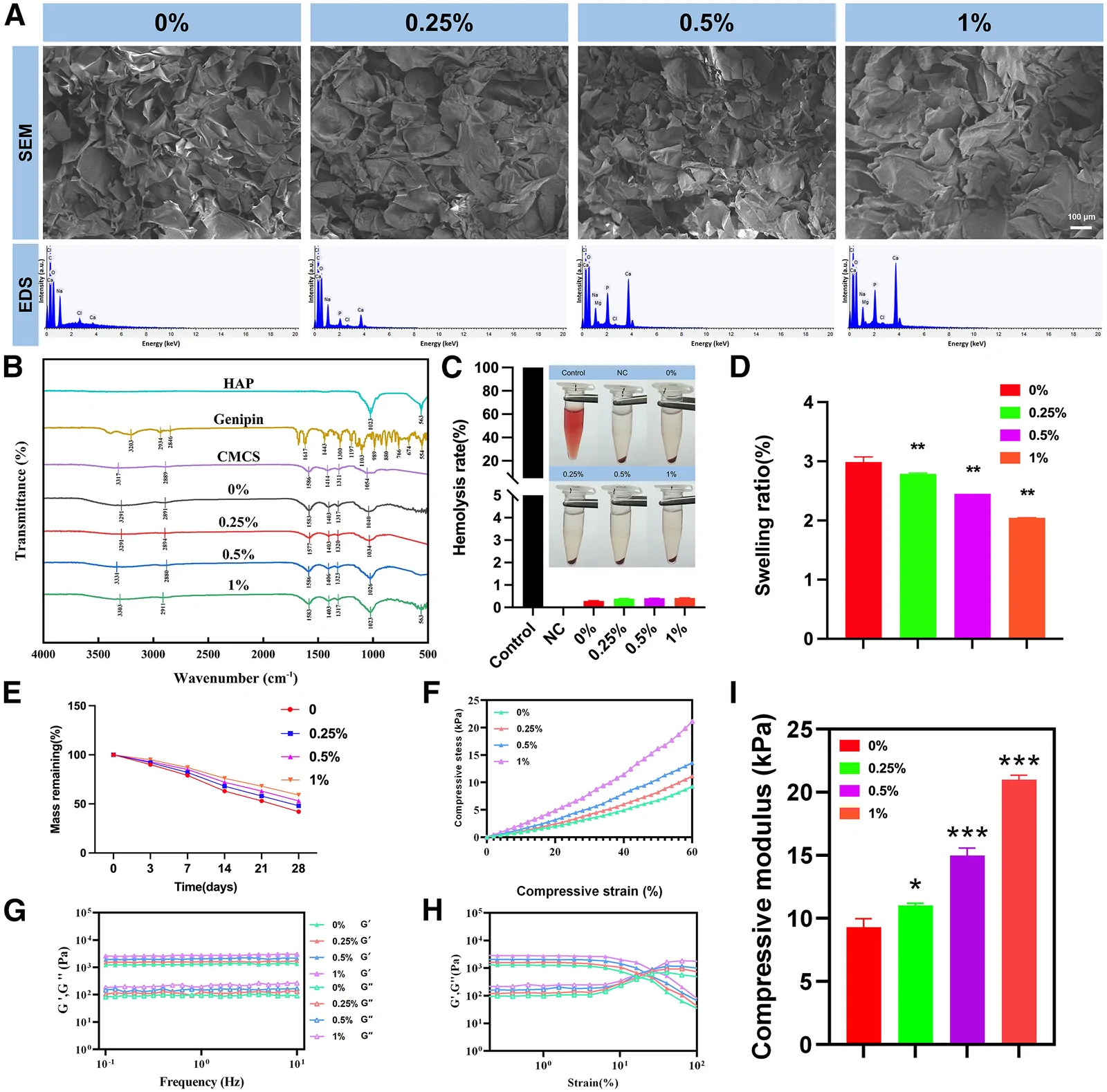
Physicochemical and mechanical characterization of CMCS/GP/nHA composite hydrogels (A) SEM images and corresponding EDS spectra of hydrogels with increasing nHA content (0%, 0.25%, 0.5%, and 1%). (B) FT-IR spectra showing characteristic peaks of CMCS, GP, and nHA interactions. (C) Hemolysis assay demonstrating blood compatibility of the hydrogels. (D) Swelling behavior of hydrogels in deionized water. (E) *In vitro* degradation profiles of hydrogels in PBS over 28 days. (F) Compressive stress-strain curves of hydrogels with different nHA contents. (G) Frequency sweep rheological analysis showing storage modulus (G′) and loss modulus (G″). (H) Strain sweep rheological analysis of the hydrogels. (I) Quantitative analysis of compressive modulus. Quantitative data are presented as mean ± standard deviation (SD), *n* = 3. ∗*p* < 0.05, ∗∗*p* < 0.01, and ∗∗∗*p* < 0.001 versus the 0% nHA group; ns indicates no significant difference. Scale bars, 100 μm.

Scanning electron microscopy (SEM) analysis revealed a uniform and interconnected porous microarchitecture across all groups, which is favorable for cell attachment, proliferation, and nutrient transport (Figure 1A). Energy-dispersive X-ray spectroscopy (EDS) spectra demonstrated a progressive increase in Ca and P signals with increasing nHA loading, confirming successful mineral incorporation and suggesting enhanced osteoconductive potential of the composite hydrogels (Figure 1A). To further verify the mineral ion-releasing behavior of the composite hydrogels, Ca^2+^ and PO_4_^3-^ release profiles were monitored over 28 days. The 0% nHA hydrogel showed negligible ion release, whereas nHA-containing hydrogels exhibited sustained Ca^2+^ and PO_4_^3-^ release in a concentration-dependent manner (Figures S1D and S1E). Notably, the 1% nHA group displayed the highest Ca^2+^ and PO_4_^3-^ release among all groups, supporting its stronger mineral-related bioactivity and microenvironmental regulation capacity.

Fourier transform infrared spectroscopy (FT-IR) further verified chemical structural modifications after crosslinking and nHA addition (Figure 1B). Enhanced characteristic peaks associated with –NH and –COO^−^ groups indicated the formation of stable covalent interactions between CMCS and GP. Meanwhile, the increasing intensity of phosphate-related absorption bands with higher nHA concentrations confirmed effective integration of the inorganic phase, which likely contributed to higher hydrogen bonding density and improved structural compactness.

The maximum swelling ratio decreased with elevated nHA content (Figure 1D), presumably due to the increased crosslinking density and reduced pore size induced by mineral reinforcement. Consistently, *in vitro* degradation in phosphate-buffered saline (PBS) exhibited a controlled and gradual profile in all groups, with slower degradation observed in hydrogels containing higher nHA levels (Figure 1E), indicating improved mechanical stability suitable for long-term support during bone repair.

Hemolysis testing confirmed that all hydrogels exhibited excellent blood compatibility without inducing detectable hemolytic reactions, demonstrating favorable biosafety properties (Figure 1C). Mechanical characterization further confirmed the reinforcing effect of nHA incorporation within the CMCS/GP hydrogel network. Compressive stress-strain analysis showed that the resistance of the hydrogels to compressive deformation gradually increased with increasing nHA content (Figure 1F). Rheological frequency sweep analysis demonstrated that the storage modulus (G′) was higher than the loss modulus (G″) across the tested frequency range, indicating the formation of a stable elastic hydrogel network (Figure 1G). Strain sweep analysis further showed that the hydrogels maintained gel-like behavior within the linear viscoelastic region, while higher nHA incorporation improved the mechanical robustness of the network (Figure 1H). Consistently, quantitative analysis of the compressive modulus revealed a concentration-dependent increase, with the 1% nHA hydrogel exhibiting the highest compressive modulus among all groups (Figure 1I). These results indicate that nHA incorporation not only enhances osteoconductive potential but also improves the mechanical stability of CMCS/GP hydrogels, which is beneficial for maintaining scaffold integrity during bone repair.

Collectively, these results confirm that CMCS/GP/nHA composite hydrogels possess optimized physicochemical stability, controllable degradation behavior, improved rheological and compressive mechanical properties, and enhanced osteoconductive potential, establishing a robust foundation for their subsequent biological performance in bone regeneration.

### *In vitro* cytocompatibility evaluation of the composite hydrogels

To assess the biosafety and cell-supportive properties of the hydrogels, third-passage BMSCs isolated from rat femurs and tibias were characterized by flow cytometry. The cells exhibited high expression levels of CD44 and CD90 and negative expression for endothelial marker CD31 and hematopoietic marker CD45, confirming a typical mesenchymal phenotype (Figure S1B).

**Figure 2:**
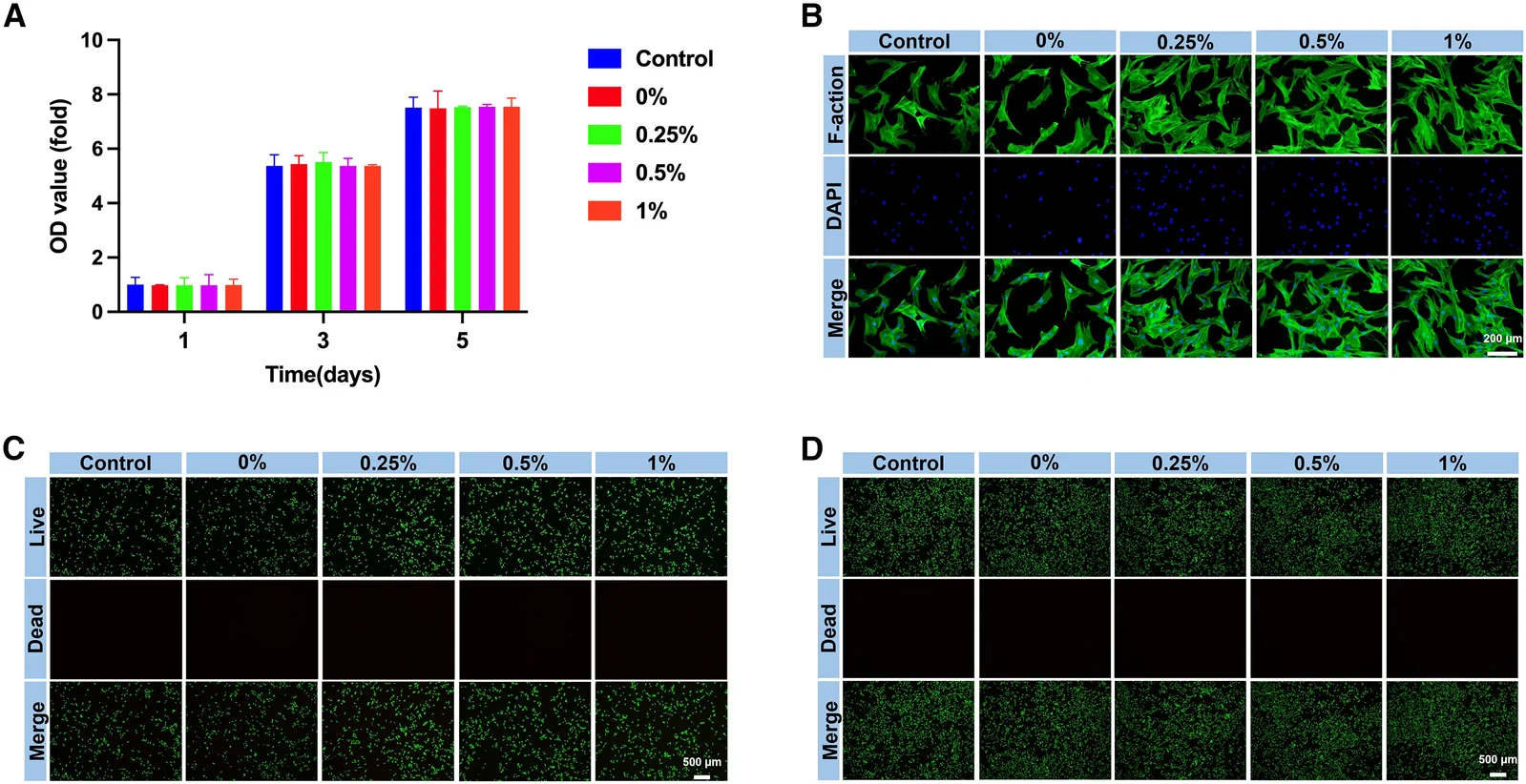
*In vitro* cytocompatibility and cell adhesion on CMCS/GP/nHA hydrogels (A) CCK-8 assay showing BMSC proliferation on hydrogels over 5 days. (B) FITC-phalloidin staining of F-actin cytoskeleton in BMSCs cultured on hydrogels for 24 h. (C and D) Live/dead staining of BMSCs co-cultured with hydrogels for 24 h and 72 h, respectively (green, live cells; red, dead cells). Quantitative data are presented as mean ± SD, *n* = 3. ns indicates no significant difference compared with the blank control group. Scale bars, 200 μm (B), 500 μm (C and D).

Cell counting kit-8 (CCK-8) assays demonstrated that BMSCs cultured on all hydrogel groups exhibited sustained proliferation over 1, 3, and 5 days, with no significant difference compared with the blank control group (Figure 2A), indicating negligible cytotoxicity. Live/dead staining further verified the excellent cell viability, showing predominantly viable cells with strong green fluorescence and only a minimal number of red-stained dead cells (Figures 2C and 2D).

Fluorescein isothiocyanate (FITC)-phalloidin staining revealed well-spread cytoskeletal organization of BMSCs adhered on the hydrogel surfaces. Notably, increasing nHA incorporation promoted more extensive cell spreading and elongation (Figure 2B), suggesting that mineral reinforcement creates a more favorable microenvironment for mechanotransduction and osteogenic commitment.

Collectively, these findings confirm that CMCS/GP/nHA hydrogels exhibit excellent cytocompatibility and provide a supportive substrate for BMSC adhesion and proliferation, establishing a solid foundation for their subsequent osteogenic performance.

### Osteogenic differentiation of BMSCs induced by composite hydrogels

**Figure 3:**
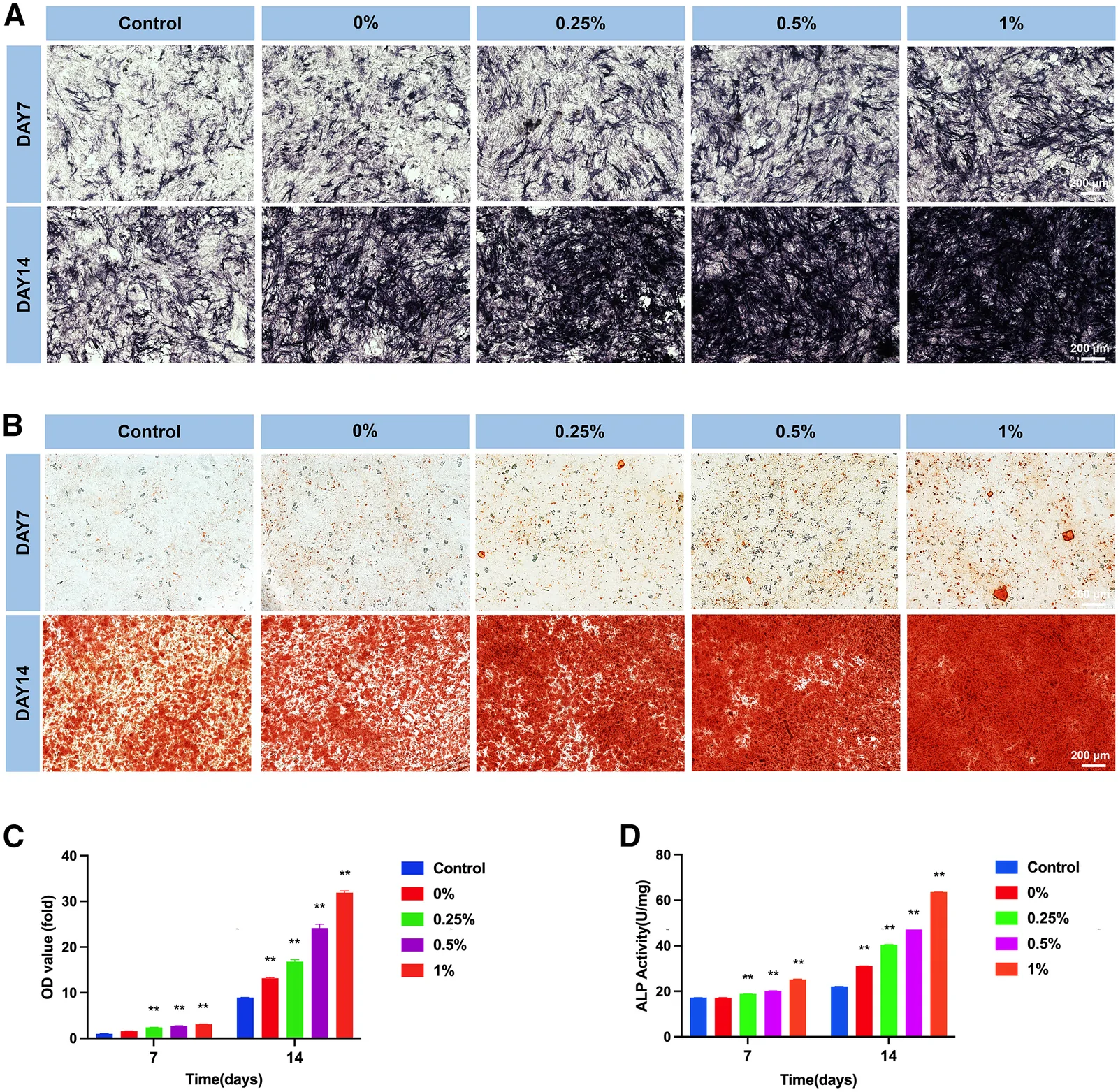
Osteogenic differentiation of BMSCs induced by CMCS/GP/nHA hydrogels (A) ALP staining of BMSCs after 7 days of osteogenic induction. (B) ARS staining of mineralized nodules after 14 days of culture. (C) Quantitative analysis of ARS staining intensity. (D) Quantitative ALP activity assay. Quantitative data are presented as mean ± SD, *n* = 3. ∗*p* < 0.05 and ∗∗*p* < 0.01 versus the blank control group at the same time point. Scale bars, 200 μm.

To evaluate the osteoinductive capability of the composite hydrogels, both early and late-stage osteogenic markers of BMSCs were systematically assessed. ALP staining revealed that all hydrogel groups promoted early osteogenic differentiation, with intensified staining corresponding to increased nHA concentration (Figure 3A). Quantitative ALP activity analysis demonstrated consistent results, confirming enhanced osteogenic induction with higher nHA loading (Figure 3D).

Alizarin Red S (ARS) staining was performed to examine extracellular matrix mineralization during late-stage differentiation. The composite hydrogel groups exhibited substantially increased calcium nodule deposition compared with the blank control, with the 1% nHA group showing the strongest mineralization (Figure 3B). Quantification further indicated a dose-dependent enhancement of mineral deposition by nHA incorporation (Figure 3C).

**Figure 4:**
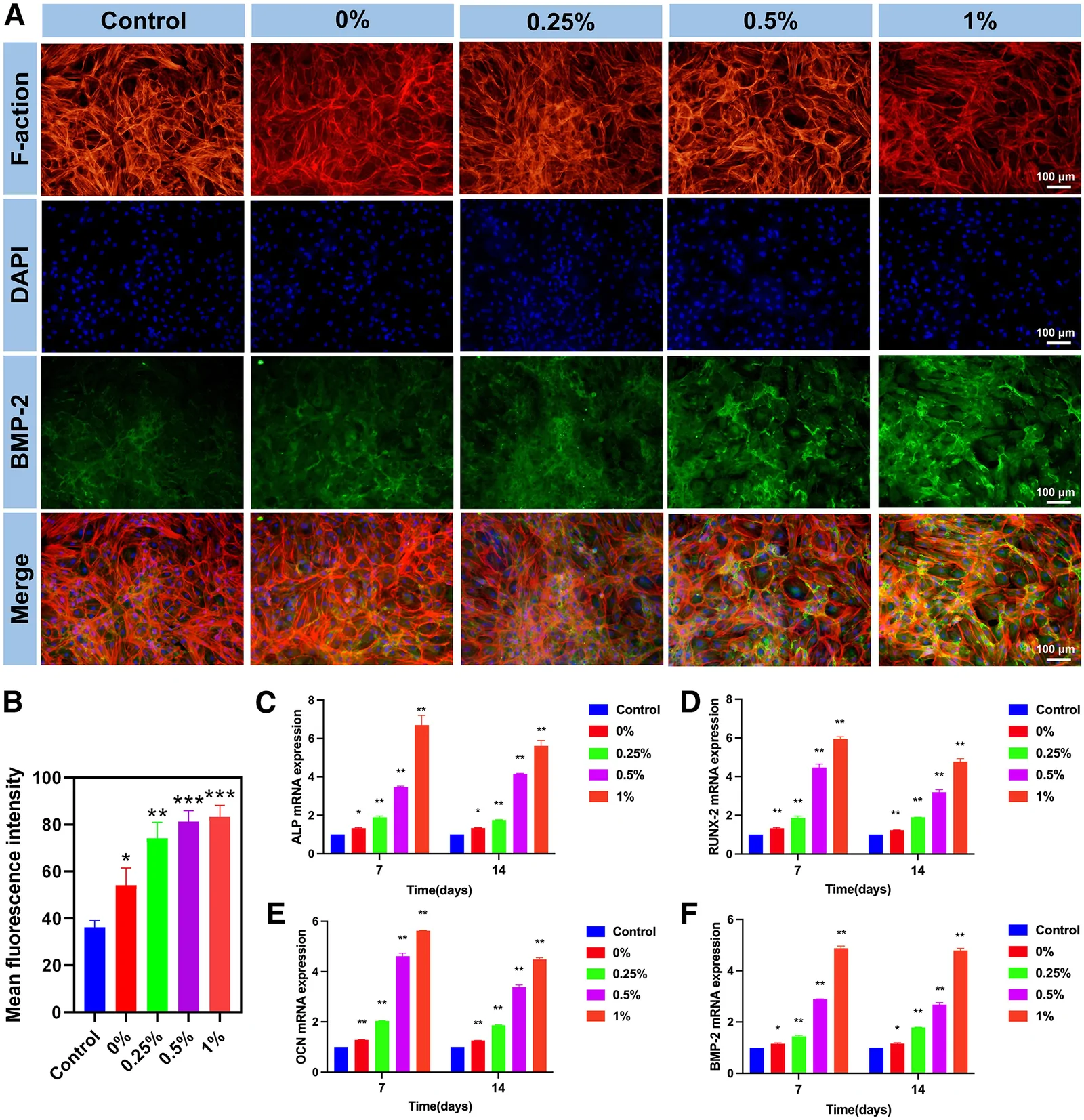
Molecular analysis of osteogenic gene and protein expression in BMSCs cultured on composite hydrogels (A) Immunofluorescence staining of BMP-2 expression in BMSCs after 48 h of transwell co-culture with hydrogels. (B) Quantitative analysis of BMP-2 fluorescence intensity. RT-qPCR analysis of osteogenic gene expression after 7 and 14 days of osteogenic induction: (C) ALP, (D) RUNX2, (E) OCN, and (F) BMP-2. Quantitative data are presented as mean ± SD, *n* = 3. ∗*p* < 0.05 and ∗∗*p* < 0.01 versus the blank control group. Scale bars, 100 μm.

Since BMP-2 plays an essential role in bone regeneration, immunofluorescence staining was used to evaluate its protein expression in BMSCs after 48 h of transwell co-culture with the hydrogels. A marked upregulation of BMP-2 was observed in BMSCs co-cultured with nHA-containing hydrogels, especially in groups containing higher nHA concentrations, suggesting activation of osteogenic signaling pathways (Figure 4A). Quantitative analysis of BMP-2 fluorescence intensity further confirmed that BMP-2 expression increased in a concentration-dependent manner after treatment with nHA-containing hydrogels (Figure 4B).

To further elucidate the molecular mechanisms underlying the promoted osteogenesis, real-time quantitative polymerase chain reaction (RT-qPCR) was conducted to detect the expression of osteogenesis-related genes. ALP, the hallmark of early osteogenic commitment (Figure 4C), and RUNX2, the master transcription factor driving BMSC osteogenic lineage progression (Figure 4D), were significantly upregulated in hydrogel-treated cells. Moreover, osteocalcin (OCN), a late-stage mineralization-associated protein (Figure 4E), and BMP-2, a critical osteogenic growth factor (Figure 4F), also exhibited markedly elevated expression. Notably, each gene displayed a positive correlation with nHA concentration, indicating regulatory effects across multiple osteogenic stages.

Taken together, these findings demonstrate that CMCS/GP/nHA hydrogels significantly enhance osteogenic differentiation of BMSCs by stimulating early lineage commitment (RUNX2, ALP), promoting matrix maturation and mineralization (OCN), and activating osteogenic growth factor signaling (BMP-2). The synergistic contributions from the bioactive CMCS matrix, biomimetic mineral phase (nHA), and stable GP-crosslinked structure collectively endow the hydrogels with potent osteoinductive properties.

### *In vivo* bone regeneration performance of the composite hydrogels

**Figure 5:**
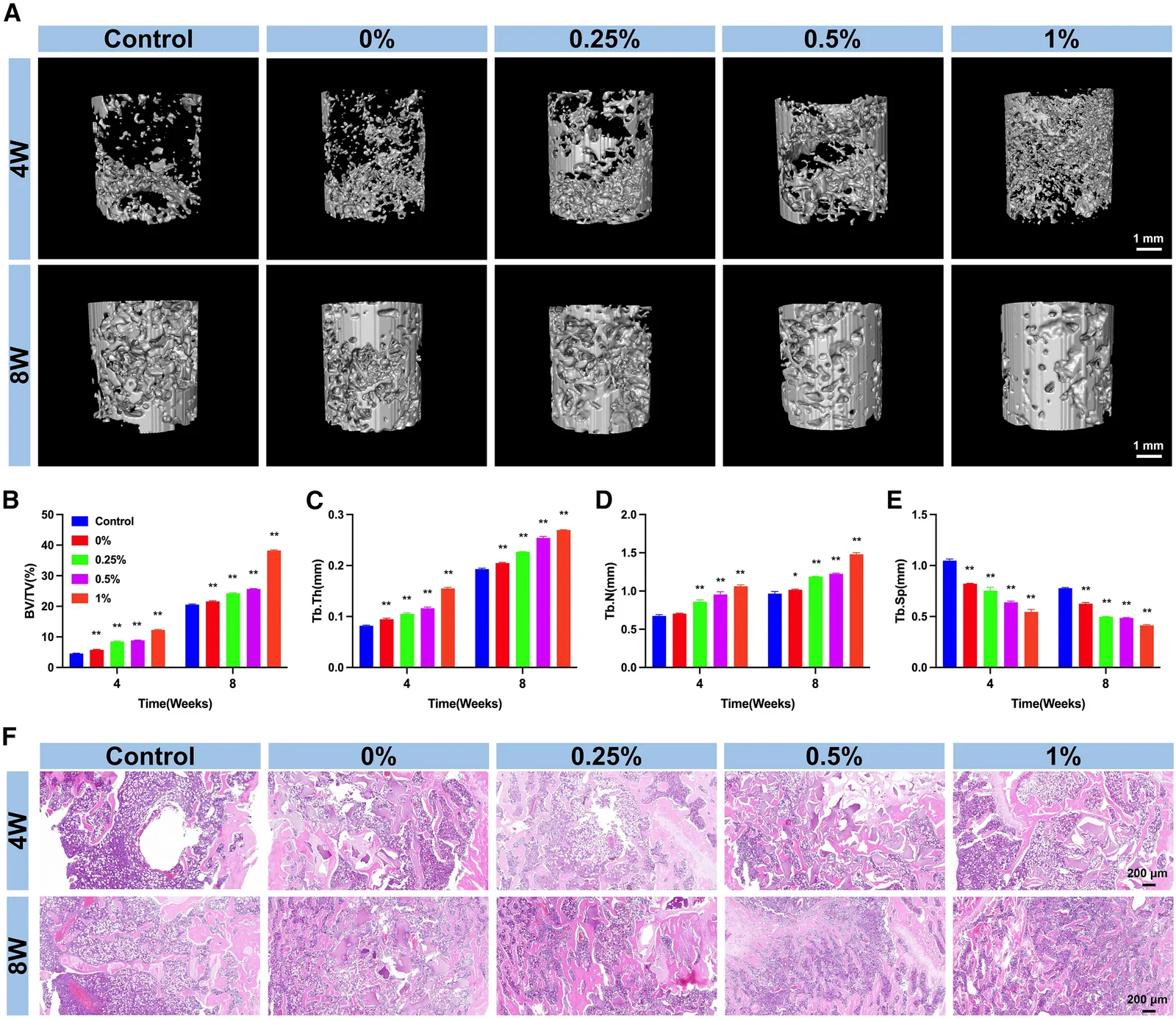
*In vivo* bone repair in a standardized rat distal femoral condyle defect model assessed by micro-CT imaging and histological staining (A) Representative 3D reconstructed micro-CT images of femoral defects at 4 and 8 weeks post-implantation. Quantitative micro-CT parameters: (B) bone volume fraction (BV/TV), (C) trabecular thickness (Tb.Th), (D) trabecular number (Tb.N), and (E) trabecular separation (Tb.Sp). (F) Representative H&E staining images of the bone defect regions at 4 and 8 weeks post-implantation. Quantitative data are presented as mean ± SD, *n* = 3. ∗*p* < 0.05 and ∗∗*p* < 0.01 versus the blank control group at the same time point. Scale bars, 1 mm (A), 200 μm (F).

To investigate the *in vivo* regenerative efficacy of the composite hydrogels, a standardized distal femoral condyle defect model was established in rats, followed by implantation of the respective hydrogel scaffolds. Bone regeneration was evaluated via micro-computed tomography (micro-CT) three-dimensional reconstruction at 4 and 8 weeks post-surgery (Figure 5A). The results revealed markedly enhanced new bone formation in all hydrogel-treated groups compared with the blank control group, with the 1% nHA hydrogel demonstrating the most pronounced repair effect.

Quantitative micro-CT analysis showed a significant increase in bone volume fraction (BV/TV), trabecular thickness (Tb.Th), and trabecular number (Tb.N) in the hydrogel groups, indicating elevated bone mass and improved trabecular architecture (Figures 5B–5D). In addition, trabecular separation (Tb.Sp) was significantly reduced (Figure 5E), confirming denser bone trabeculas and more complete defect filling in the implanted groups. Histological evaluation by hematoxylin and eosin (H&E) staining further confirmed the micro-CT findings (Figure 5F). At 4 weeks, the blank control group showed limited tissue filling within the defect region, whereas hydrogel-treated groups exhibited increased cellular infiltration and newly formed bone-like tissue. At 8 weeks, more mature and continuous regenerated tissue was observed in the hydrogel-treated groups, particularly in the 1% nHA group, which showed more complete defect filling and denser bone-like structure. These histological observations further support the ability of CMCS/GP/nHA hydrogels to promote bone defect repair.

**Figure 6:**
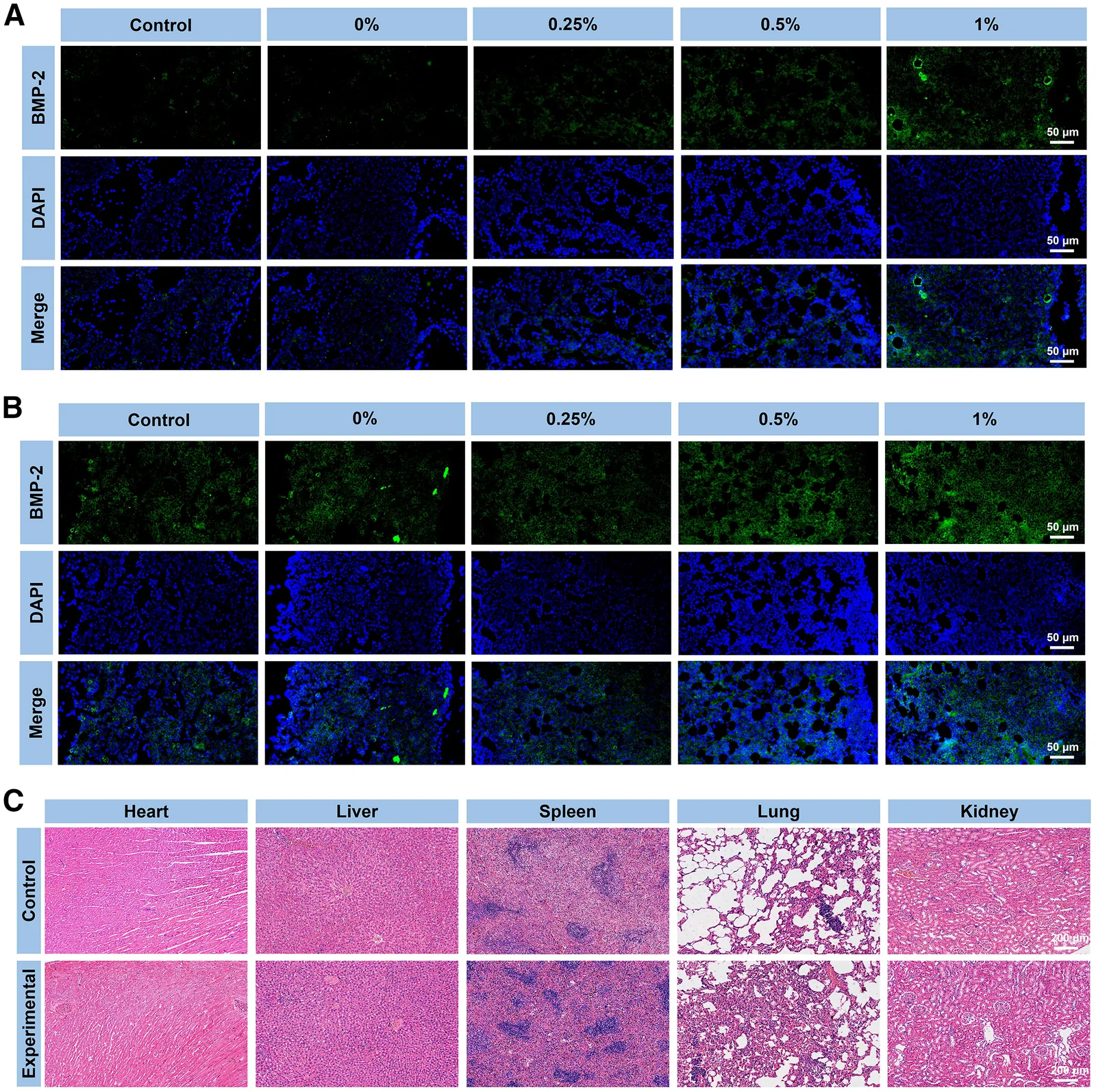
Immunofluorescence and histological evaluation of *in vivo* bone repair and systemic biocompatibility (A and B) Immunofluorescence staining of BMP-2 expression in defect regions at (A) 4 weeks and (B) 8 weeks post-implantation. (C) H&E staining of major organs (heart, liver, spleen, lung, and kidney) from rats implanted with hydrogels for 8 weeks, demonstrating no pathological alterations. Scale bars, 500 μm (A and B), 200 μm (C).

Immunofluorescence staining further demonstrated that BMP-2 expression was significantly upregulated at both 4 and 8 weeks in the hydrogel-treated defects, supporting the restoration of osteogenic signaling and functional bone tissue formation (Figures 6A and 6B). Meanwhile, histological evaluation of major organs showed no pathological alterations in the hydrogel-treated animals compared with controls, indicating favorable systemic biosafety of the implanted materials (Figure 6C).

Overall, both imaging and histological findings demonstrate that the CMCS/GP/nHA composite hydrogels significantly accelerated bone repair in the rat distal femoral condyle defect model, with the 1% nHA formulation exhibiting the most favorable regenerative performance among the tested groups.

## Discussion

Achieving precise coordination among material structure, biological activity, and regenerative performance remains a key challenge in the development of BTE scaffolds. In this study, we adopted a biomimetic design strategy to fabricate CMCS/nHA/GP composite hydrogels and explored their potential in promoting bone defect repair. Our findings reveal a synergistic enhancement in both cellular regulation and *in vivo* bone regeneration, which can be attributed to the complementary functions of the hydrogel components and structural characteristics.

CMCS, as a natural polysaccharide with excellent biocompatibility, provides an extracellular matrix (ECM)-like environment supporting cell adhesion, spreading, and metabolic activity. Meanwhile, nHA—an analog of the inorganic mineral phase of bone—contributes not only to fundamental osteoconduction but also to microenvironmental ionic modulation through Ca^2+^ and PO_4_^3−^ release. Previous studies have demonstrated that nHA can trigger integrin-mediated cytoskeletal remodeling and activate RUNX2-driven osteogenic lineage commitment.^52^ This aligns with our observations of improved cellular spreading and upregulation of osteogenic markers in BMSCs cultured on the composite hydrogels. The ion release kinetics further supported this interpretation. The sustained and concentration-dependent release of Ca^2+^ and PO_4_^3−^ from nHA-containing hydrogels confirmed that nHA could continuously modulate the local mineral ionic microenvironment. In particular, the 1% nHA group showed the highest Ca^2+^ and PO_4_^3−^ release within the tested range, which may partially explain its superior osteogenic performance.

The osteogenic effects exhibited a clear concentration-dependent trend in response to nHA incorporation, emphasizing the need for a balance between osteoconductive potential and hydrogel network stability. In this study, the 1% nHA formulation achieved the most favorable outcomes among the tested groups, likely because it provided sufficient mineral-related bioactivity, enhanced ion exchange, and improved local mineralization capacity while maintaining appropriate porosity, swelling behavior, degradation kinetics, and mechanical integrity. However, this result should not be interpreted as evidence that further increasing nHA content would continuously enhance osteogenesis. During preliminary formulation optimization, higher nHA contents resulted in reduced dispersion stability and visible precipitation, suggesting impaired nanoparticle distribution and compromised hydrogel homogeneity. Excessive nHA loading may also interfere with GP-mediated network formation, reduce pore interconnectivity, impair nutrient diffusion, and weaken cell-material interactions. Therefore, 1% nHA represents an optimized concentration within the current CMCS/GP system, balancing hydrogel processability, structural stability, and osteogenic bioactivity. The mechanical data further confirmed that nHA acted as a reinforcing inorganic phase within the CMCS/GP hydrogel network. The increased compressive modulus and the predominance of G′ over G″ suggested improved mechanical stability and elastic behavior after nHA incorporation. This moderate mechanical reinforcement may help maintain scaffold integrity within the defect site and provide a more favorable microenvironment for bone regeneration. In addition to structural support, the altered mechanical properties of the hydrogels may also contribute to BMSC osteogenic differentiation through mechanotransduction-related regulation. Matrix stiffness and viscoelasticity are important physical cues that can be sensed by cells through integrin-mediated focal adhesion formation. The enhanced compressive modulus and elastic-dominant behavior of nHA-containing hydrogels may promote cytoskeletal organization, intracellular tension, and downstream mechanosensitive signaling, including FAK/ROCK/ERK and YAP/TAZ-related pathways.^53^^,^^54^ This interpretation is consistent with the enhanced F-actin organization and cell spreading observed in BMSCs cultured on nHA-containing hydrogels. Therefore, the osteogenic enhancement observed in this study may result from the synergistic effects of nHA-derived mineral ion release and stiffness-associated mechanotransduction. Recent studies have demonstrated that nHA/polymer composite systems can effectively improve scaffold bioactivity and bone-regenerative performance.^55^ For example, nHA combined with CS- or other natural polymer-based matrices has been reported to enhance osteoconductivity, mineral deposition, cell adhesion, and osteogenic differentiation.^56^ Similarly, nHA-containing composite hydrogels or 3D-printed polymer scaffolds have shown improved mechanical properties and accelerated bone repair by integrating the osteoconductive mineral phase with a cell-supportive polymer network.^57^ However, many of these systems are based on a fixed homogeneous nHA content or require additional structural reinforcement, bioactive factors, or drug-loading strategies to enhance regeneration. Compared with these approaches, the present study highlights a graded low-dose nHA-loading design within a GP-crosslinked CMCS hydrogel matrix. This design enables systematic optimization of mineral content while avoiding excessive nHA-induced aggregation or network instability. Moreover, GP crosslinking provides a relatively low-toxicity strategy to stabilize the CMCS network, improve scaffold integrity, and support long-term bone repair. Therefore, the main contribution of this work lies not merely in incorporating nHA into a polymer hydrogel, but in combining graded mineral loading with GP-mediated crosslinking to achieve a balanced improvement in processability, degradation behavior, mechanical properties, and osteogenic bioactivity.

It should be noted that the *in vivo* model used in this study was a standardized rat distal femoral condyle defect model rather than a strict segmental critical-sized defect model. This model was selected because it provides a reproducible metaphyseal bone defect environment for evaluating scaffold-mediated bone repair while avoiding the mechanical instability associated with large segmental femoral defects. Nevertheless, given the relatively small defect diameter, possible spontaneous repair cannot be completely excluded. Therefore, the *in vivo* findings should be interpreted as evidence that CMCS/GP/nHA hydrogels accelerate bone repair in a rat femoral condyle defect model. Future studies using larger non-healing defects or mechanically stabilized segmental defect models are required to further validate their therapeutic potential for clinically relevant large bone defects. Notably, the composite hydrogels exhibited intrinsic osteoinductive capabilities beyond mere defect filling. Increased BMP-2 expression *in vivo* suggests activation of endogenous bone repair signaling, a critical advantage for clinical translation compared with passive scaffolds requiring exogenous factors. Although BMP-2-mediated osteogenic activation was the main focus of the present study, the angiogenic microenvironment is also essential for successful bone regeneration. Bone repair requires coordinated vascular invasion and osteogenic differentiation, because newly formed vessels provide oxygen, nutrients, osteoprogenitor cells, and angiocrine signals for mineralized tissue formation. The porous and hydrated CMCS/GP network may support nutrient exchange and cellular infiltration, whereas nHA-derived Ca^2+^ and PO_4_^3−^ may contribute to local ionic modulation and matrix mineralization.^58^ These features may indirectly create a microenvironment favorable for osteogenesis-angiogenesis coupling. However, direct angiogenesis-related evidence, such as CD31 immunofluorescence staining, VEGF expression analysis, vWF staining, or microvessel density quantification, was not included in the present study. This limitation should be addressed in future studies to clarify whether CMCS/GP/nHA hydrogels directly promote vascularized bone regeneration.

The proposed strategy also demonstrates high scalability. Osteogenic-angiogenic coupling, widely recognized as essential for large-segment bone regeneration, may be achieved by tailoring the nanoparticle composition, modulating pore architecture, or incorporating pro-angiogenic agents. Additionally, integrating controlled-release profiles or stimuli-responsive functionalities may enable applications in complex pathological environments such as infectious or osteoporotic bone defects.

### Limitations of the study

Despite these promising findings, several limitations must be acknowledged. First, although the rat distal femoral condyle defect model provided a reproducible environment for evaluating scaffold-mediated bone repair, it may not fully represent a strict non-healing critical-sized defect or clinically complex large bone defects. Second, the present study mainly focused on BMP-2-related osteogenic activation, whereas direct angiogenesis-related assessments were not performed. Third, although 1% nHA exhibited the best performance among the tested formulations, a broader range of nHA concentrations and improved dispersion strategies should be further investigated to determine the upper loading threshold. Fourth, although H&E staining was added to evaluate the general histological repair of the bone defect region, Masson staining was not performed in the present study. Future studies should include collagen-specific histological staining, such as Masson trichrome staining, to further assess collagen deposition and matrix maturation during bone regeneration. Fifth, long-term mechanical stability and validation in diseased or load-bearing defect models are needed to further support clinical translation. Sixth, the present study used only male rats in the *in vivo* experiments, and thus sex-related differences in bone regeneration were not analyzed.

Collectively, this work introduces a structurally tunable, highly biomimetic composite hydrogel capable of stimulating endogenous bone repair. Future efforts focusing on mechanical reinforcement, vascularization improvement, and validation in clinically relevant diseased models are expected to accelerate its translation toward therapeutic applications for critical-sized bone defects.

## Resource availability

### Lead contact

Further information and requests for resources and reagents should be directed to and will be fulfilled by the lead contact, Lanfeng Huang (hlf@jlu.edu.cn).

### Materials availability

This study did not generate new unique reagents.

### Data and code availability

• All data reported in this study will be shared by the lead contact upon request.
• This study does not report original code.
• Any additional information required to reanalyze the data reported in this study is available from the lead contact upon request.

## Acknowledgments

The authors acknowledge financial support from the Special Project for Medical and Health Talent of Jilin Province (2024WSZX-B13).

## Author contributions

Xudong Li: writing – original draft; C.Y.: conceptualization and writing – review and editing; Xin Li: investigation; L.L.: conceptualization; Y.L.: validation; T.L.: methodology and validation; Q.G.: methodology; L.H.: supervision, funding acquisition, conceptualization, and writing – review and editing.

## Declaration of interests

The authors declare no competing interests.

## STAR★Methods

### Key resources table

**Table:** 

REAGENT or RESOURCE	SOURCE	IDENTIFIER
**Chemicals, peptides, and recombinant proteins**		

Genipin (GP)	Aladdin	Cat#G105502
α-Ketoglutaric acid	Aladdin	Cat#K104336
Dimethyl sulfoxide (DMSO)	Sigma-Aldrich	Cat#D4540
Dexamethasone	Sigma-Aldrich	Cat#D4902
Paraformaldehyde	Sigma-Aldrich	Cat#P6148
Triton X-100	Sigma-Aldrich	Cat#T8787
Bovine serum albumin (BSA)	Sigma-Aldrich	Cat#A2153
DAPI	Sigma-Aldrich	Cat#D9542

**Antibodies**		

Anti-BMP-2 antibody (rabbit polyclonal)	Abcam	Cat# ab14933; RRID: AB_2243574

**Assay kits**		

ALP staining kit (BCIP/NBT)	Beyotime	Cat#C3206
Alizarin Red S staining kit	Cyagen	Cat#ALIR-10001
CCK-8 Cell Counting Kit-8	Dojindo	Cat#CK04

**Critical commercial assays**		

QuantiTect SYBR Green PCR Kit	Qiagen	Cat#204143
PrimeScript RT reagent Kit	Takara	Cat#RR037A
TRIzol Reagent	Invitrogen	Cat#15596026

### Experimental model and study participant details

#### Animals

All animal procedures were approved by the Institutional Animal Care and Use Committee of Jilin University (Approval No. SYT2024036) and conducted in accordance with established ethical guidelines. Male Sprague–Dawley rats (8–10 weeks old, weighing 250–300 g) were used to establish the distal femoral condyle defect model. Animals were housed under standard conditions with a 12 h light/dark cycle and *ad libitum* access to food and water.

#### Primary BMSCs

Primary bone marrow mesenchymal stem cells (BMSCs) were isolated from the femurs and tibias of 4-week-old male Sprague–Dawley rats using a standard bone marrow flushing method. Cells were cultured in DMEM/F12 medium supplemented with 10% fetal bovine serum (FBS) and 1% penicillin–streptomycin at 37°C in a 5% CO_2_ humidified atmosphere. The culture medium was refreshed every 2–3 days. Cells at passage 3 were used for all subsequent experiments and authenticated via flow cytometric detection of surface markers (CD44^+^CD90^+^CD31^−^CD45^−^). Cell cultures were routinely tested and confirmed negative for mycoplasma contamination using a Mycoplasma Detection Kit (Beyotime, China) according to the manufacturer’s instructions.

### Method details

#### Preparation and characterization of CMCS/nHA composite hydrogels

Composite hydrogels containing different mass fractions of nano-hydroxyapatite (nHA) were prepared through a chemical crosslinking method. Briefly, 1.5 g of carboxymethyl chitosan (CMCS) was dissolved in 100 mL of deionized water under magnetic stirring and left to stand for 12 h to ensure complete dissolution, resulting in a 1.5% (w/v) CMCS solution. Subsequently, 0, 0.025, 0.05, and 0.1 g of nHA were ultrasonically dispersed into 10 mL of CMCS solution to obtain final nHA mass fractions of 0%, 0.25%, 0.5%, and 1%, respectively.

Genipin (GP) was dissolved in dimethyl sulfoxide (DMSO) to prepare a 1% (w/v) GP solution, which was then added to the CMCS/nHA mixtures to initiate crosslinking. The mixtures were incubated at 37°C for 24 h until complete formation of stable hydrogels.

The surface morphology and pore structure of the hydrogels were observed using scanning electron microscopy (SEM, Sigma 300, Carl Zeiss, Germany). The chemical functional groups and interaction between CMCS, nHA, and GP were analyzed using Fourier transform infrared spectroscopy (FT-IR, TENSOR 27, Bruker, Germany).

#### Swelling behavior and *in vitro* degradation assays

The swelling behavior of the hydrogels was evaluated to determine their water absorption capacity. Briefly, freeze-dried hydrogel samples from each group were accurately weighed and recorded as W_0_. The samples were then immersed in deionized water at room temperature. At predetermined intervals, the hydrogels were removed, gently blotted to remove excess surface water, and weighed to obtain the maximum swollen weight W_max_. The equilibrium swelling ratio was calculated using the following equation: Swelling ratio(%) = (W_max_-W_0_)/W_0_×100%

The *in vitro* degradation behavior was assessed by monitoring the weight loss of hydrogels over time. Freeze-dried samples were weighed as W_0_ and incubated in phosphate-buffered saline (PBS, pH 7.4) at 37°C in a shaking incubator (100 rpm). The PBS solution was refreshed every two days. At predetermined time points (3, 7, 14, 21, and 28 days), hydrogels were removed, freeze-dried, and weighed as W_t_. The remaining mass was calculated using: Remaining mass(%) = W_t_/W_0_×100%. To evaluate the ion release behavior of the hydrogels, samples from each group were immersed in PBS at 37°C. At predetermined time points, the supernatants were collected for analysis. The concentrations of released Ca^2+^ and PO_4_^3-^ were measured using corresponding assay kits according to the manufacturer’s instructions. The release profiles were plotted as a function of time. All measurements were performed in triplicate.

#### Rheological and compressive mechanical characterization

The rheological properties of the hydrogels were evaluated using a rotational rheometer. Briefly, hydrogel samples from each group were placed between parallel plates, and frequency sweep tests were performed within the linear viscoelastic region to determine the storage modulus (G′) and loss modulus (G″). Strain sweep tests were further conducted to evaluate the strain-dependent viscoelastic behavior and network stability of the hydrogels.

The compressive mechanical properties of the hydrogels were assessed using a universal mechanical testing machine. Cylindrical hydrogel samples from each group were compressed at a constant loading rate, and compressive stress–strain curves were recorded. The compressive modulus was calculated from the linear region of the stress–strain curve. All tests were performed on at least three independent samples for each group.

#### Hemocompatibility evaluation of the composite hydrogels

The hemocompatibility of the hydrogels was assessed using a standard hemolysis assay. Whole blood was collected from healthy rats using anticoagulant-treated tubes, and erythrocytes were isolated by centrifugation followed by removal of fibrin-containing components. A 4% (v/v) red blood cell (RBC) suspension was prepared using phosphate-buffered saline (PBS). Triton X-100 was used as the positive control, while PBS served as the negative control.

Hydrogel extracts were prepared by immersing sterilized samples in PBS at 37°C for 24 h. Equal volumes of RBC suspension were mixed with the hydrogel extracts and control solutions, followed by incubation at 37°C for 1 h. After incubation, the mixtures were centrifuged, and the absorbance of the supernatants was measured at 540 nm to determine the level of hemoglobin release. Hemolysis percentage was calculated relative to the controls.

#### *In vitro* biocompatibility evaluation of the composite hydrogels

Bone marrow mesenchymal stem cells (BMSCs) were freshly harvested from rats using a standard bone marrow flushing method. The cells were cultured to passage 3 and characterized by flow cytometry to confirm their mesenchymal stem cell phenotype.

To assess cytocompatibility, CCK-8 assays were performed to evaluate cell proliferation. BMSCs were seeded in 24-well plates at a density of 1 × 10^4^cells per well. Sterilized hydrogels with different nHA mass fractions (0%, 0.25%, 0.5%, and 1%) were placed in Transwell inserts, and a blank control (without hydrogel) was included. Cell viability was measured at 1, 3, and 5 days by adding CCK-8 reagent and quantifying absorbance using a microplate reader.

Live/dead staining was conducted under the same culture conditions as described above. At 24 h and 72 h, calcein-AM and propidium iodide (PI) staining were performed to visualize viable and dead cells, followed by observation under a fluorescence microscope.

FITC–phalloidin staining was used to assess cell morphology and cytoskeletal organization. After co-culturing cells with hydrogels for 24 h, cells were fixed and stained with FITC–phalloidin to label filamentous actin (F-actin). Fluorescence images were captured using a fluorescence microscope to evaluate cell spreading behavior.

#### *In vitro* osteogenic induction of the composite hydrogels

BMSCs were cultured as described above. To induce osteogenic differentiation, the medium was supplemented with dexamethasone (100 nM), β-glycerophosphate (10 mM), and ascorbic acid (50 μg/mL).

To assess early- and late-stage osteogenesis, BMSCs cultured in the lower chamber of the Transwell system were stained at days 7 and 14, respectively. For alkaline phosphatase (ALP) staining, cells were fixed with 4% paraformaldehyde and stained using an ALP staining kit (Beyotime Biotechnology, China) according to the manufacturer’s protocol. For mineralization analysis, Alizarin Red S (ARS) staining was performed using commercial reagents (Cyagen, China). After staining, excess dye was removed and cells were washed thoroughly with PBS. Images were captured and quantified using ImageJ software.

For BMP-2 expression evaluation, BMSCs were seeded in the lower chamber of 24-well Transwell plates at a density of 2.5 × 10^4^ cells per well. Sterilized hydrogels with different nHA mass fractions were placed in the upper Transwell inserts and co-cultured with BMSCs for 48 h. Cells were then fixed with 4% paraformaldehyde, incubated with a polyclonal anti-BMP-2 primary antibody, followed by Alexa Fluor 647-conjugated anti-rabbit IgG secondary antibody (1:1000, Abcam, China). Fluorescence images were acquired using a fluorescence microscope. BMP-2 fluorescence intensity was quantified using ImageJ software and expressed as mean fluorescence intensity.

To further investigate the regulatory effects of the composite hydrogels on osteogenic differentiation, the expression of osteogenesis-related genes including RUNX2, ALP, OCN, and BMP-2 was examined using real-time quantitative PCR (RT-qPCR). After 7 and 14 days of co-culture, total RNA was extracted from BMSCs using TRIzol reagent and reverse transcribed into cDNA. RT-qPCR was performed using a QuantiTect SYBR Green kit (Qiagen, Germany) on a QuantStudio 3 thermal cycler (Applied Biosystems, USA). Gene expression levels were calculated using the 2^−ΔΔCt^ method, with GAPDH used as the internal control.

#### Animal experiments and *in vivo* bone regeneration assessment

All animal procedures were approved by the Institutional Animal Care and Use Committee of Jilin University (Approval No. SYT2024036) and conducted in accordance with established ethical guidelines. A standardized distal femoral condyle defect model was established in Sprague–Dawley rats. Fifty male rats underwent bilateral femoral surgery. Anesthesia was induced using 2–3% isoflurane, and two cylindrical defects measuring 3 mm in diameter and 3 mm in depth were created in the femoral condyle using a saline-cooled drill. Composite hydrogels were implanted into the defect sites accordingly. Postoperative antibiotic treatment was administered for the first three days. At 4 and 8 weeks after surgery, three rats from each group were randomly selected for further evaluation.

To precisely quantify bone regeneration, high-resolution micro-computed tomography (micro-CT) was performed on femurs fixed in 4% paraformaldehyde. Scans were acquired at 48 kV tube voltage, 200 μA current, and a voxel size of 18 μm. Three-dimensional reconstructions were performed using multimodal visualization software, and bone morphometric parameters were quantified, including bone volume fraction (BV/TV), trabecular thickness (Tb.Th), trabecular number (Tb.N), and trabecular separation (Tb.Sp). Additionally, immunofluorescent staining for BMP-2 was conducted to evaluate osteogenic protein expression around the regenerated bone.

For histological evaluation of bone defect repair, femoral condyle specimens were decalcified, dehydrated, embedded in paraffin, and sectioned. The sections were stained with hematoxylin and eosin (H&E) to observe tissue regeneration, cellular infiltration, and defect filling within the bone defect region.

To assess systemic biocompatibility, rats were euthanized with CO_2_ inhalation at 8 weeks post-implantation. Major organs including the heart, liver, spleen, lungs, and kidneys were harvested and fixed in 4% paraformaldehyde. Hematoxylin and eosin (H&E) staining was performed to examine potential pathological alterations in the organ tissues.

### Quantification and statistical analysis

All data were analyzed using OriginPro 2024 (OriginLab, USA) and GraphPad Prism 9.0 software (GraphPad Software, USA). Results are presented as mean ± standard deviation (SD). Statistical comparisons were performed using Student’s t test and one-way or two-way analysis of variance (ANOVA), followed by Student–Newman–Keuls post hoc tests where appropriate. A value of *p* < 0.05 was considered statistically significant, and *p* < 0.01 was regarded as highly significant.
