# Supplementary material for: Biofunctional carboxymethyl chitosan hydrogels with nano-hydroxyapatite gradients accelerate bone defect healing
Source: iScience. 2026 Aug 14;29(9):117200. doi: 10.1016/j.isci.2026.117200 (PMC13503097; doi:10.1016/j.isci.2026.117200)
Supplement: Supplementary file 1 [file mmc1.pdf]

iScience, Volume *29*

Supplemental information

Biofunctional carboxymethyl chitosan hydrogels with nano-hydroxyapatite gradients accelerate bone defect healing

Xudong Li, Chengzhe Yang, Xin Li, Lanxin Li, Youbin Li, Tengyue Liu, Qiang Gao, and Lanfeng Huang

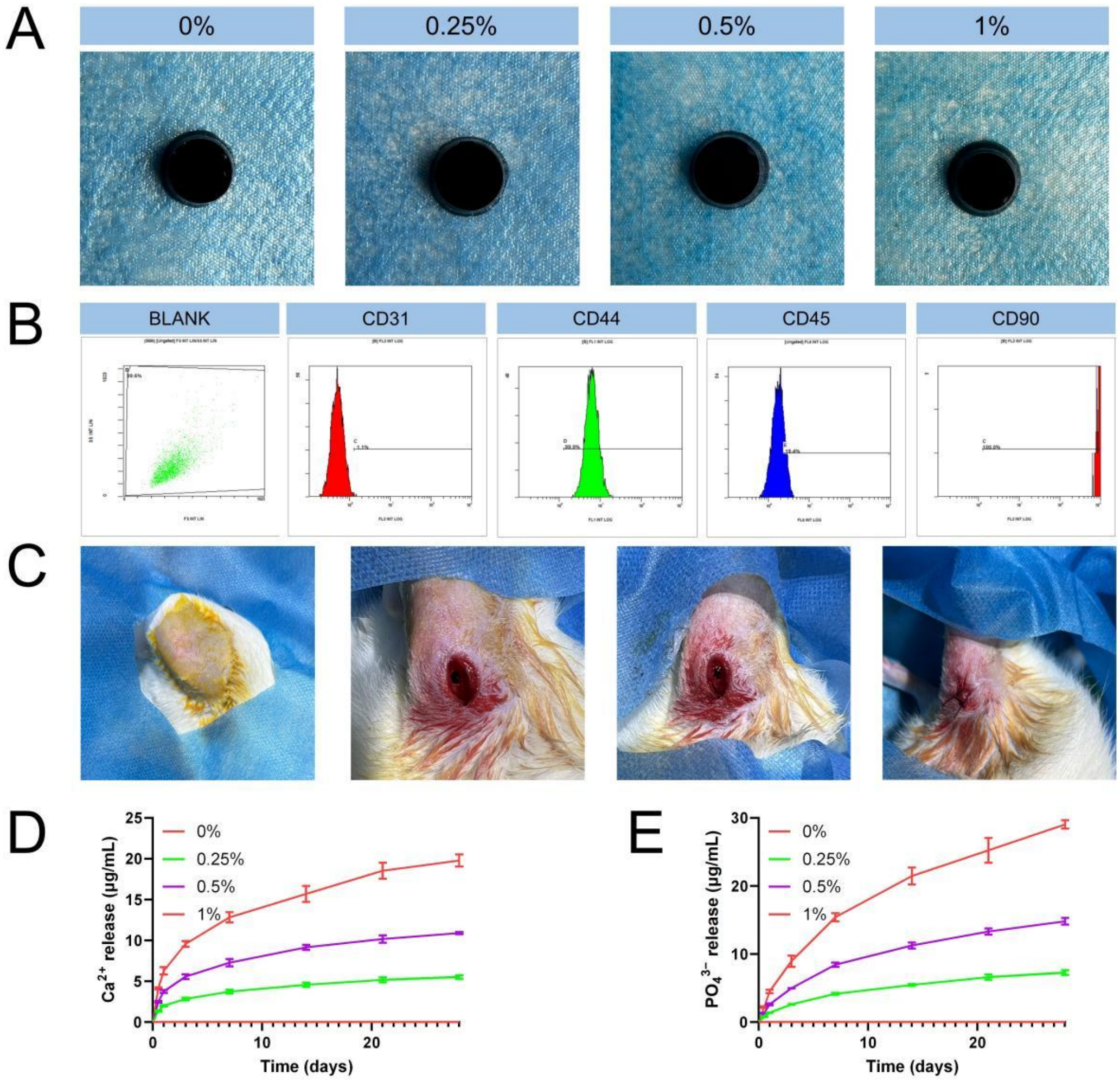

Supplementary Figure 1. Additional characterization of CMCS/GP/nHA hydrogels and the rat defect model.

(A) Representative photographs of hydrogels with different nHA contents.
(B) Flow cytometric characterization of isolated rat BMSCs, including CD31, CD44, CD45, and CD90 expression.
(C) Representative intraoperative photographs of the rat distal femoral condyle defect model and hydrogel implantation.
(D) Ca^{2+} release profiles of hydrogels with different nHA contents over 28 days.
(E) PO_4^{3-} release profiles of hydrogels with different nHA contents over 28 days.
